# Hyperhomocysteinemia is independently associated with albuminuria in the population-based CoLaus study

**DOI:** 10.1186/1471-2458-11-733

**Published:** 2011-09-26

**Authors:** Franziska Marti, Peter Vollenweider, Pedro-Manuel Marques-Vidal, Vincent Mooser, Gérard Waeber, Fred Paccaud, Murielle Bochud

**Affiliations:** 1 University Institute of Social and Preventive Medicine (IUMSP), University of Lausanne and Centre Hospitalier Universitaire Vaudois (CHUV), 1005 Lausanne, Switzerland; 2 Department of Internal Medicine, Centre Hospitalier Universitaire Vaudois (CHUV), Lausanne, Switzerland; 3 CardioMet, University of Lausanne, Switzerland; 4 Department of pathology and laboratory medicine, Centre Hospitalier Universitaire Vaudois (CHUV), Lausanne, Switzerland

## Abstract

**Background:**

Increased serum levels of homocysteine and uric acid have each been associated with cardiovascular risk. We analyzed whether homocysteine and uric acid were associated with glomerular filtration rate (GFR) and albuminuria independently of each other. We also investigated the association of *MTHFR *polymorphisms related to homocysteine with albuminuria to get further insight into causality.

**Methods:**

This was a cross-sectional population-based study in Caucasians (*n *= 5913). Hyperhomocysteinemia was defined as total serum homocysteine ≥ 15 μmol/L. Albuminuria was defined as urinary albumin-to-creatinine ratio > 30 mg/g.

**Results:**

Uric acid was associated positively with homocysteine (r = 0.246 in men and r = 0.287 in women, *P *< 0.001). The prevalence of albuminuria increased across increasing homocysteine categories (from 6.4% to 17.3% in subjects with normal GFR and from 3.5% to 14.5% in those with reduced GFR, *P *for trend < 0.005). Hyperhomocysteinemia (OR = 2.22, 95% confidence interval: 1.60-3.08, *P *< 0.001) and elevated serum uric acid (OR = 1.27, 1.08-1.50, per 100 μmol/L, *P *= 0.004) were significantly associated with albuminuria, independently of hypertension and type 2 diabetes. The 2-fold higher risk of albuminuria associated with hyperhomocysteinemia was similar to the risk associated with hypertension or diabetes. *MTHFR *alleles related to higher homocysteine were associated with increased risk of albuminuria.

**Conclusions:**

In the general adult population, elevated serum homocysteine and uric acid were associated with albuminuria independently of each other and of renal function.

## Background

Homocysteine, a sulfur containing amino acid involved in methionine metabolism, is a potential atherogenic molecule and is considered as an independent risk factor for cardiovascular disease (CVD) [1-3]. Serum levels of total homocysteine (tHcy) are elevated in patients with chronic kidney disease (CKD) and, more generally, in patients with reduced renal function [1,4]. Several studies have shown that elevated tHcy levels are associated with atherothrombotic disease in coronary, cerebral and peripheral arteries [3,5]. A similar phenotype is associated with CKD [6,7]. Similar pathological changes are observed in glomerular injury and tHcy-induced vascular damages, such as endothelial dysfunction, cell proliferation, increased oxidative stress and prothrombotic state [8,9]. As a consequence, high tHcy levels may potentially induce renal injury via direct action on kidney cells, rather than only result as a consequence of impaired renal function. In other words, tHcy could be involved as a cause of renal atherosclerosis and renal damage, leading to a vicious cycle with accelerated deterioration of renal function.

To date, tHcy has been associated with microalbuminuria in population-based studies [1,10,11] and in diabetic patients [10,12,13]. The population-based Hoorn study, which included subjects aged 50 to 75 years, found that baseline tHcy was associated with microalbuminuria not only at baseline [10], but also after a mean follow-up of 6.1 years, independently of renal function [11]. In the National Health and Nutrition Examination Survey 1991-1994, the largest population-based study available so far (3387 subjects aged ≥ 40 years), elevated tHcy was associated with albuminuria independently of vitamin B status [1]. However, it remains unclear whether tHcy causes albuminuria or is merely the consequence of reduced renal function, which is itself reflected by albuminuria [14]. As albuminuria is an established marker of kidney damage in diabetic and nondiabetic individuals and a strong predictor of cardiovascular morbidity and mortality [15,16], a better understanding of the determinants of albuminuria is therefore of great interest.

Serum uric acid (SUA) is associated with increased risk of incident kidney disease [17], and it also predicts mortality in patients with CKD [18]. Several studies have shown a link between SUA concentrations and tHcy [19,20]. The extent to which tHcy and SUA may independently influence renal function and renal damage is not known. Although the association of renal function and tHcy levels is well-known in selected clinical samples [21], only few large scale population-based data have been published so far [1,10]. To our knowledge none of the latter investigated the role of SUA in this context.

The aim of this study was to evaluate the association between elevated tHcy and the presence of impaired renal function, expressed as either decreased glomerular filtration rate (GFR) or albuminuria in a large population-based sample in Switzerland. We also investigated the potential modifying effect of SUA on these relationships. In order to evaluate whether the association between tHcy and albuminuria could be causal, we looked at the association between albuminuria and the methylenetetrahydrofolate reductase (*MTHFR) *gene polymorphisms strongly correlated with tHcy.

## Methods

### Study population

The CoLaus Study has been described previously [22]. Briefly, a simple non-stratified random sample of 35% of the overall population of the city of Lausanne, Switzerland, aged 35-75 years (*n *= 56,694) was drawn. Inclusion criteria were: a) written informed consent; b) age between 35 and 75 years; c) Caucasian origin. Out of the 6188 participants, we excluded 145 subjects because of various missing values, leaving 5913 subjects for the present analysis.

The study was approved by the ethical committee of the Faculty of Medicine of the University of Lausanne (Switzerland) and the research conformed to the Helsinki Declaration. All participants provided written informed consent.

### Assessment process and data collection

Participants attended the outpatient clinic of the University Hospital Center of Lausanne (CHUV) in the morning after an overnight fast. BMI was defined as weight in kilograms divided by height in meters squared (kg/m^2^). Blood pressure was measured three times on the left arm using a clinically validated automatic oscillometric device [23]Hypertension was defined as a mean systolic blood pressure ≥ 140 mmHg and/or a diastolic blood pressure ≥ 90 mmHg and/or presence of antihypertensive drug treatment.

### Biological data

A venous blood sample (50 ml) was collected from each participant under fasting conditions. The analytical procedures for biological markers and clinical chemistry methods have been described previously [22]. Hyperhomocysteinemia was defined as a tHcy level ≥ 15 μmol/L [1]. Diabetes was defined as a fasting blood glucose ≥ 7 mmol/l and/or presence of any antidiabetic drug (including insulin).

A spot urine sample was collected for the assessment of urinary creatinine and albumin, and the ACR was calculated. Albuminuria was defined as a value of ACR above 30 mg/g, which includes microalbuminuria (ACR 30-300 mg/g) and macroalbuminuria (ACR > 300 mg/g) [24]. Glomerular filtration rate (GFR) was estimated by the simplified MDRD prediction equation: GFR (ml/min/1.73 m2) = 186 × (S_Cr_)^-1.154 ^× (Age)^-0.203 ^× (0.742 if female), where S_Cr _is serum creatinine concentration in mg/dL and age is in years [25]. We also conducted sensitivity analyses using the Cockcroft-Gault formula to estimate creatinine clearance (C_Cr_): C_Cr _= (140 - age) × (weight)/72 × S_Cr _x (0.83 if female), where S_Cr _is in mg/dL, weight in kg and age in years [26].

### Questionnaire data

Trained health professionals used standardized questionnaires on socio-demographic characteristics and lifestyle factors, such as tobacco, alcohol consumption and vitamin intake. Participants were asked whether they were taking vitamin supplements on a regular basis and if yes, which type of vitamins. No specific information allowing differentiating intakes of vitamin B6, B11 and B12 was collected. For the purpose of the present analyses, smokers were defined as current smokers and non-smokers as never- or ex-smokers. Alcohol consumption was converted in standard units per day.

### Genotyping and quality controls

Nuclear DNA was extracted from whole blood for entire genome scan analysis. Genotyping was performed using the Affymetrix 500 K chip, as recommended by the manufacturer. Individuals with less than 95% genotyping efficiency overall (or < 90% efficiency on either array, n = 399), and individuals with possible gender inconsistencies (n = 5), were removed. Monomorphic single nucleotide polymorphisms (SNPs), SNPs with less than 70% genotyping efficiency, SNPs with minor allele frequency < 2% and SNPs not in Hardy-Weinberg proportions were excluded from analyses, leaving at the end 4155 participants for the present genetic analysis. We analyzed the 38 genotyped SNPs located within and around (500 kb) the *MTHFR *gene for association with tHcy, using multiple linear regression. Because of its known clinical interest, the functional non-synonymous *MTHFR C677T *polymorphism (*rs1801133) *[27] was also included in the analysis, even though it was only imputed. Imputation was done based on HapMap Phase II haplotypes using the IMPUTE software, as previously described [28]. Three of the genotyped SNPs were strongly associated with tHcy levels (*rs9651118*, R^2 ^= 0.51%, *P *= 2 × 10^-7 ^; *rs1321073*, R^2 ^= 0.45%, *P *= 7 × 10^-7 ^and *rs34175640*, R^2 ^= 0.45%, *P *= 7 × 10^-7^), as was the imputed *C677T *polymorphism (*rs1801133*, R^2 ^= 0.57%, *P *= 9 × 10^-7^). Out of the three genotyped SNPs, two were in perfect linkage disequilibrium (*rs1321073 *and *rs34175640*), leaving only three partially independent SNPs for the present analysis: *rs1321073, rs9651118 and rs1801133*.

### Statistical analyses

Statistical analyses were performed using Stata 10.0 (Stata Corp, College Station, USA). Results were expressed as mean ± standard deviation (sd), median ± interquartile range or median ± standard error (se). For comparison of data between groups, we used a t-test for continuous variables and a chi square test for dichotomous variables. We used a non parametric test (nptrend in Stata) to test for linear trend across selected categories whenever appropriate. We analyzed the association between tHcy and SUA using a spearman rank correlation test. For descriptive purposes, we split tHcy into 4 categories (tHcy < 9, 9 ≤ tHcy < 12, 12 ≤ tHcy < 15, and tHcy ≥ 15 μmol/L), as previously published [1]. MDRD was dichotomized into < 90, and ≥ 90 mL/min/1.73 m^2 ^categories. We used median regression to analyze the association of MDRD with tHcy (dependent variable), while adjusting for the potential confounding effect or effect modification of selected covariates. A *P*-value < 0.05 was considered as statistically significant for mean effects. We systematically tested all two-way interactions of MDRD with all other covariates in the model. We used a Bonferroni-corrected *P*-value to select which interaction terms to keep in the model (e.g. if 10 interaction terms were tested, we used 0.005 as a cut-off *P*-value, etc). To keep the models hierarchically sound, covariates included in a significant interaction term were kept as main effects in the model. To ease interpretation of the significant interaction terms on tHcy, we also conducted stratified analyses by age (below vs above the mean age of 53 years) and by serum uric acid strata (below vs above the mean level of 321 μmol/L). We used multiple logistic regression to analyze the association of hyperhomocysteinemia with dichotomized albuminuria. We systematically tested all two-way interactions between hyperhomocysteinemia or sex, on the one side, and all other covariates in the model, on the other side, and used a Bonferroni-corrected *P*-value to select interaction terms to be kept in the model. We analyzed the association of the SNPs with albuminuria using logistic regression with an additive mode of action. The corresponding *P*-values are not adjusted for multiple testing.

## Results

Among the 5913 participants aged 35-75 years, with a mean (± sd) age at 53 years (± 11 years), 52.5% were females. Table 1 displays participants' characteristics stratified by sex. Men were significantly younger than women, reported higher alcohol consumption and lower intake of B vitamins. Men had higher body mass index (BMI), blood pressure, Modification of Diet in Renal Disease (MDRD, estimation of GFR), fasting plasma glucose, and SUA levels. Men also had a higher prevalence of type 2 diabetes, smoking, hypertension and albuminuria than women. Women had significantly lower tHcy levels (9.3 (2.7) μmol/L vs. 10.9 (3.1) μmol/L, *P *< 0.001) and prevalence of hyperhomocysteinemia (4.13% vs. 10.7%, *P *< 0.001) than men. tHcy levels were positively correlated with SUA in men (r = 0.246, *P *< 0.001) and in women (r = 0.287, *P *< 0.001).

**Table 1:** Participants' characteristics by sex

Characteristics	Men (*n *= 2766)	Women (*n *= 3147)
Age (years)	52.4 (10.7)	53.4 (10.7)
Alcohol consumption (units/day)	1.49 (1.57)	0.57 (0.80)
Smokers (%)	28.3	24.9
Intake of B vitamins (%)	5.8	8.9
Use of diuretics (%)	6.9	6.4
Body mass index (kg/m^2^)	26.6 (4.0)	25.1 (4.8)
Type 2 diabetes (%)	9.2	3.5
Hypertension (%)	41.5	29.8
Systolic blood pressure (mmHg)	132.0 (16.5)	124.7 (18.2)
Diastolic blood pressure (mmHg)	81.2 (10.7)	77.5 (10.5)
Hyperhomocysteinemia (%)	10.7	4.13
**Serum measurements**		
Total homocysteine (μmol/L)	10.9 (3.1)	9.27 (2.7)
Creatinine (μmol/L)	88.1 (15.6)	72.0 (11.6)
MDRD ^c ^(ml/min/1.73 m^2^) ^b^	85.9 (20.0)	79.3 (18.6)
Uric acid (μmol/L)	360.4 (74.8)	269.9 (66.4)
Total cholesterol (mmol/L)	5.56 (1.04)	5.61 (1.03)
HDL-cholesterol (mmol/L)	1.44 (0.36)	1.81 (0.42)
Fasting glucose (mmol/L)	5.78 (1.23)	5.34 (0.99)
**Urine measurements**		
Albumin to creatinine ratio (mg/g) ^a^	4.56 (5.26)	5.58 (5.88)
Albuminuria ^c ^(%)	6.9	5.4

Data are expressed as mean (standard deviation), n (%) or median ^a ^(interquartile range).

^b ^Modification of Diet in Renal Disease, estimation of glomerular filtration rate

^c ^urinary albumin-to-creatinine ratio > 30 mg/g

**Figure 1:**
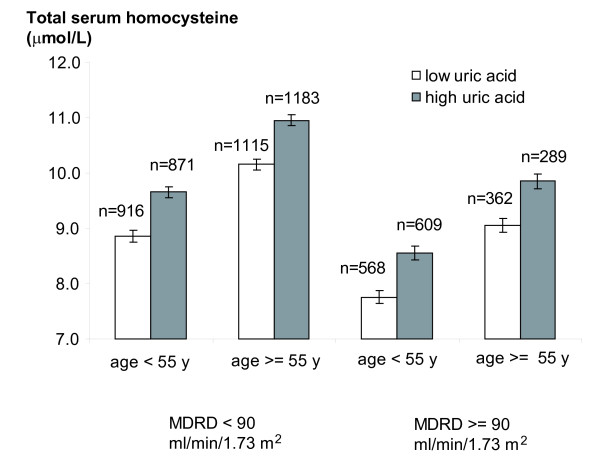
**Total serum homocysteine levels by categories of MDRD, age and uric acid levels**. Bars (whiskers) are medians (se) adjusted for sex, categories of age, MDRD and serum uric acid levels. *P*-values for age, MDRD and serum uric acid categories < 0.001 from a multiple median regression using total serum homocysteine as the dependent variable. MDRD, Modification of Diet in Renal Disease, estimation of glomerular filtration rate.

In multiple regression analysis using tHcy as the dependent variable, the association of MDRD with tHcy was modified by age and SUA (Table 2, *P *for interaction < 0.001) after adjusting for major confounding factors. When conducting stratified analyses, the regression coefficient for MDRD on tHcy was -0.043 [95%CI: -0.050; -0.036] and -0.060 [-0.066; -0.054] in the younger and older age groups, respectively, and -0.042 [95%CI: -0.048; -0.036] and -0.057 [-0.064; -0.049] for the low and high uric acid groups, respectively. To ease the interpretation of these findings, Figure 1 shows the adjusted median tHcy levels by MDRD and age groups. Within each group, tHcy was more elevated in the high SUA category than in the low SUA category. Sensitivity analyses using the Cockcroft-Gault formula led to similar results.

**Table 2:** Determinants of total serum homocysteine

	β-Coefficient	**95% CI **^d^	
Sex (0 = men, 1 = women)	-1.341	-1.540	-1.141 ^e^
Age (years)	0.136	0.097	0.176 ^e^
Alcohol consumption (units/day)	0.098	0.034	0.162 ^e^
Smoking status	0.468	0.291	0.645 ^e^
Intake of B vitamins	-0.616	-0.904	-0.328 ^e^
Use of diuretics	0.394	0.072	0.716 ^e^
Body mass index (kg/m^2^)	-0.019	-0.038	0.001
Systolic blood pressure (mmHg)	0.006	0.001	0.011 ^e^
Total serum cholesterol (mmol/L)	0.030	-0.046	0.105
Plasma fasting glucose (mmol/L)	-0.023	-0.096	0.050
Serum uric acid (μmol/L)	0.014	0.009	0.019 ^e^
MDRD ^a ^(ml/min/1.73 m2)	0.037	0.010	0.064 ^e^
MDRD × age ^b^	-0.0010	-0.0015	-0.0006 ^e^
MDRD × uric acid ^c^	-0.00011	-0.00016	-0.00005 ^e^

^a ^Modification of Diet in Renal Disease, estimation of glomerular filtration rate

^b ^Interaction between MDRD and age

^c ^Interaction between MDRD and serum uric acid

^d ^95% Confidence Interval

^e ^P value < 0.05

In multiple logistic regression, hyperhomocysteinemia (tHcy ≥ 15 μmol/L) (OR = 2.22, 95% confidence interval (CI): 1.60-3.08, *P *< 0.001) and SUA (OR = 1.27, 95%CI: 1.08-1.50, per 100 μmol/L *P *= 0.004) were significantly related with albuminuria independently of hypertension and diabetes (Table 3). The risk of albuminuria associated with hyperhomocysteinemia (OR = 2.22, 95%CI: 1.60-3.08, *P *< 0.001) was similar to that associated with hypertension (OR = 1.52, 95%CI: 1.11-2.09, *P *= 0.009) or with type 2 diabetes (OR = 1.93, 95%CI: 1.27-2.91, *P *= 0.002). We found no significant interaction between hyperhomocysteinemia and any of the covariate in the model, including SUA. Further adjustment for ACE inhibitor and angiotensin receptor blocker treatment did not substantially modify the association of hyperhomocysteinemia with albuminuria (OR = 2.18, 95%CI: 1.57-3.04), while decreasing the association of hypertension with albuminuria (OR = 1.39; 95%CI: 0.99-1.96).

**Table 3:** Determinants of albuminuria

**Albuminuria **^**a**^	Odds Ratio	**95% CI **^**c**^	*P*-values
Smoking status (1 = current smoker, 0 = non-smoker)	1.35	1.05 - 1.74	0.018
Intake of B vitamins (1 = intake, 0 = no intake)	1.09	0.70 - 1.69	0.700
MDRD ^b ^(ml/min/1.73 m^2^)	1.005	0.997 - 1.013	0.219
Type 2 diabetes	1.93	1.27 - 2.91	0.002
Fasting glucose (mmol/L)	1.22	1.12 - 1.33	< 0.001
Hypertension	1.52	1.11 - 2.09	0.009
Systolic blood pressure (mm Hg)	1.01	1.01 - 1.02	< 0.001
Serum uric acid (per 100 μmol/L)	1.27	1.08 - 1.50	0.004
Hyperhomocysteinemia	2.22	1.60 - 3.08	< 0.001

Logistic regression model including age, sex, body mass index and age × sex interaction as additional covariates.

^a ^urinary albumin-to-creatinine ratio > 30 mg/g

^b ^Modification of Diet in Renal Disease, estimation of glomerular filtration rate

^c ^95% Confidence Interval

**Figure 2:**
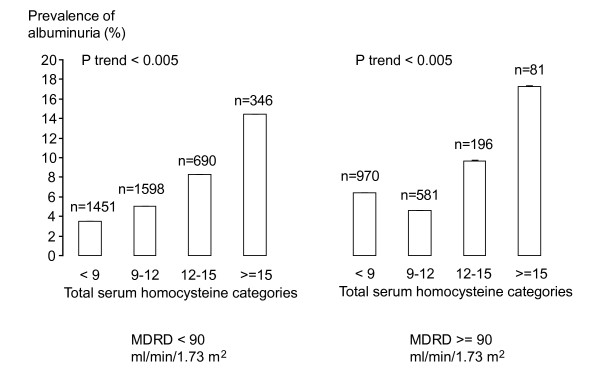
**Prevalence (95%CI) of albuminuria by categories of MDRD and total serum homocysteine**. Results were similar in men and in women. We assessed trend across total serum homocysteine categories using non-parametric test. MDRD, Modification of Diet in Renal Disease, used to estimate glomerular filtration rate.

The prevalence of albuminuria was gradually higher across increasing tHcy categories (Figure 2). A similar trend was observed in subjects with normal GFR levels (6.4%, 4.6%, 9.7% and 17.3%, *P *for trend < 0.005) and in subjects with decreased GFR levels (3.5%, 5.0%, 8.3% and 14.5%, *P *for trend < 0.005). Results were similar in men and in women (data not shown).

**Figure 3:**
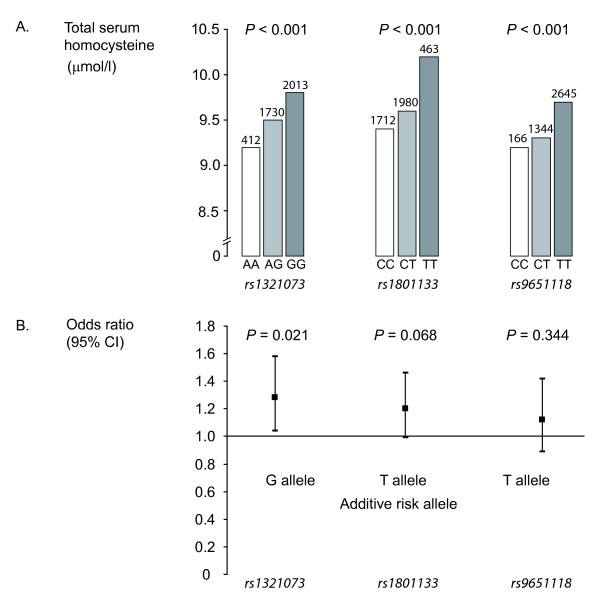
**Adjusted Odds Ratios (OR) for the risk of albuminuria by *MTHFR *gene polymorphisms**. Genetic analysis was performed in a subgroup of study (*n *= 4155). OR were adjusted for age, sex, smoking, alcohol consumption, intake of vitamin B, body mass index, hypertension, systolic blood pressure, type 2 diabetes, plasma fasting glucose, total cholesterol, serum uric acid and glomerular filtration rate. A. Median unadjusted total serum homocysteine level by genotype for each MTHFR polymorphism.B. Adjusted odds ratio for the risk of albuminuria per risk allele (additive model).

In the subsample of the study population in which genotyping was performed, we analyzed the association of three *MTHFR *polymorphisms related to tHcy levels with albuminuria (Figure 3). After adjustment for major confounding factors, the risk of albuminuria increased significantly from the AA to the GG genotype (OR = 1.28, 95%CI: 1.04-1.58 per G allele, *P *= 0.021) for the *rs1321073 *SNP. A similar trend was observed for *rs1801133 *(OR = 1.20, 95%CI: 0.99-1.46 per T allele, *P *= 0.068), but the association was not significant for *rs9651118 *(OR = 1.12, 95%CI: 0.89-1.42 per T allele, *P *= 0.344). In non-diabetic subjects, the association between albuminuria and both *rs1321073 *(OR = 1.31, 95%CI: 1.04-1.64 per G allele, *P *= 0.023) and *rs1801133 *(OR = 1.30, 95%CI: 1.05-1.61 per T allele, *P *= 0.017) was significant. A similar trend was observed for *rs9651118 *(OR = 1.11, 95%CI: 0.86-1.45 per T allele, *P *= 0.417), although not reaching significance. In type 2 diabetic subjects, these polymorphisms were associated neither with tHcy nor with albuminuria.

## Discussion

In this large population-based study, the association between elevated tHcy levels and higher prevalence of albuminuria was observed independently of GFR, reflecting the fact that this association is not simply the consequence of reduced renal function. This result is consistent with the hypothesis that homocysteine may cause renal damage. We extend previous population-based findings [1,10,11] to the age group 35-40 years. Also, the association of hyperhomocysteinemia with albuminuria was independent of hypertension, type 2 diabetes and uric acid levels. Furthermore, hyperhomocysteinemia was associated with a 2-fold higher risk of albuminuria, which was of similar magnitude to the risk of albuminuria associated with hypertension or type 2 diabetes. This result strongly suggests that hyperhomocysteinemia is an independent marker of renal dysfunction and is in line with the role of homocysteine as potential atherogenic agent and cardiovascular risk factor [2].

We observed strong associations between homocysteine levels and *MTHFR *polymorphisms, as previously reported [29-31]. This suggests that *MTHFR *genotypes related to higher homocysteine levels are associated with albuminuria in the general population, in particular in non-diabetic subjects. Because of the random assortment of alleles at the time of gamete formation, association between *MTHFR *genotypes and tHcy levels, or albuminuria, should not be biased by reverse-causality or major confounding factors. These results therefore suggest that homocysteine might be causally involved in renal damage. *MTHFR *polymorphisms have been shown to be associated with increased mortality and morbidity in healthy and CKD populations [29-31]. Some studies in diabetic patients found the *C677T *polymorphism (*rs1801133*) to be associated with renal damage [32-36], but others failed to find such association [37,38]. In this study, *rs1321073 *was significantly associated with the risk of albuminuria in the general population, independently of major confounding factors. However, this latter association was not significant in type 2 diabetic subjects, in whom no association with tHcy was observed. First of all, the number of diabetics was small. Second, since diabetes itself represents a major cause of albuminuria, the effect of homocysteine on renal damage might not be easily detectable in this subgroup, as reflected by the conflicting results reported in studies restricted to diabetic patients [32-38]. Results for the nonsynonymous *rs1801133 (C677T) *SNP, which is associated with reduced MTHFR activity [27], were quite similar. Although associated with tHcy levels, *rs9651118 *was not significantly associated with albuminuria, but the effect was nevertheless in the same direction. As each SNP only explained a tiny fraction of homocysteine variance, this study might have reduced power to detect this association.

In line with previous reports, we found a positive association between SUA and tHcy [19]. However, SUA did not modify the relationship between tHcy levels and albuminuria. This suggests that there is no strong synergistic or antagonistic effect between homocysteine and uric acid for their effect on renal damage, but rather that both factors act independently on albuminuria. Conversely, we observed an effect modification of uric acid on the known negative association between tHcy levels and GFR, independently of major confounding factors. We are not aware that the effect modification of SUA on the association between tHcy and renal function has been previously reported. Both hyperuricemia and hyperhomocysteinemia are common findings in patients with CKD. In prospective studies among CKD patients, baseline SUA and baseline tHcy levels appeared to predict overall mortality [4,18]. However the link between tHcy, SUA and decreased renal function is unclear. A possible explanation is that antioxidants, like uric acid, raise in reaction to increased oxidative stress to counteract the harmful effect of reactive oxygen species induced by elevated homocysteine [8]. Reactive oxygen species reduce glomerular blood flow and filtration rate by release of vasoconstrictors and, possibly by inactivation of NO [39].

Hyperhomocysteinemia is frequent in patients with CKD [1,4]. In this study, tHcy was higher whenever renal function was decreased. This might result, in part, from the fact that impaired renal function reduces the renal clearance of homocysteine. This could also result, in part, from impaired intrarenal homocysteine metabolism, impaired extrarenal homocysteine metabolism or both. Also the prevalence of albuminuria was gradually higher with increasing tHcy levels, independently of glomerular filtration rate, since a similar trend was observed after stratifying for normal and decreased GFR levels. Several prospective studies have shown that hyperhomocysteinemia is an independent determinant of the development of CKD and albuminuria in the general population [11,40]. Moreover, in a prospective study, kidney transplant recipients with elevated tHcy levels at baseline had a greater risk of death and kidney allograft loss [4]. These findings further support the concept that tHcy is not merely a marker, but rather a predictor of CVD. CVD currently represents the main cause of death in CKD patients, with mortality rates three to 30 times higher than expected [6,41]. As this high mortality is only partly explained by CVD risk factors, such as hypertension, diabetes, smoking and dyslipidemia, factors such as hyperhomocysteinemia and elevated uric acid levels may play a role.

Our study has several limitations. Unfortunately, we have no data on vitamin status, including folic acid, vitamin B_6_, and vitamin B_12_, but we could include data on reported vitamin B intake. However, several previous studies observed no significant interaction of vitamin B status on the association between tHcy and renal function or tHcy and cardiovascular risk [1,42]. We used estimations of GFR by the MDRD formula in this study. As MDRD has not been validated in populations with normal GFR, we also conducted sensitivity analyses using GFR estimated by the Cockcroft-Gault formula, which led to similar results. The calculated GFR and albumin-to-creatinine ratio (ACR) were based on a single measurement, as in previous population-based studies [1,10]. tHcy was also based on one single measure and is therefore insufficient to predict life-time exposure. Despite the small proportion of homocysteine variance explained, *MTHFR *polymorphisms may better reflect the life-time exposure to tHcy. However, our study also has several strengths. It is to our knowledge the largest population-based study exploring associations between homocysteine and renal function, hence with a wide external validity. A longitudinal follow-up of all participants in the CoLaus study is currently ongoing and shall provide essential data for trends over time of major cardiovascular risk factors according to baseline homocysteine levels.

## Conclusions

In conclusion, hyperhomocysteinemia was associated with higher prevalence of albuminuria in men and in women, independently of renal function. The association of hyperhomocysteinemia with albuminuria was not only independent of type 2 diabetes and hypertension, but also as strong as the one found for these two conditions, which are major causes of albuminuria. Individuals carrying *MTHFR *genotypes associated with higher tHcy concentrations were at increased risk for albuminuria. This latter finding supports the hypothesis that homocysteine causes renal damage. Further studies are needed to explore whether lowering serum homocysteine levels might prevent kidney damage. Serum uric acid was also associated with albuminuria, independently of homocysteine. Although uric acid modified the association of tHcy with GFR, no such effect modification was observed for the association between hyperhomocysteinemia and albuminuria, which speaks against a strong synergistic or antagonistic effect between homocysteine and uric acid on renal damage.

## List of abbreviations used

ACR: albumin-to-creatinine ratio; BMI: body mass index; CKD: chronic kidney disease; CVD: cardiovascular disease; GFR: glomerular filtration rate; MTHFR: methylenetetrahydrofolate reductase; SUA: Serum uric acid; tHcy: total homocysteine.

## Competing interests

The authors declare that they have no competing interests. The CoLaus study was financed in part by GlaxoSmithKline. V.M. is a consultant for GlaxoSmithKline.

## Authors' contributions

FM, conducted the literature search, the statistical analyses, intepreted the data and wrote the manuscript. MB supervised the data analysis, participated to the data interpretation and drafting of the manuscript. PMMV critically revised the manuscript for important intellectual content and participated to the data interpretation. VM, PV, FP and GW participated to the conception, design and acquisition of data and critically revised the manuscript for important intellectual content. All authors approved the final version of the manuscript.

## Pre-publication history

The pre-publication history for this paper can be accessed here:

http://www.biomedcentral.com/1471-2458/11/733/prepub
